# Threat, Coping, and Social Distance Adherence During COVID-19: Cross-Continental Comparison Using an Online Cross-Sectional Survey

**DOI:** 10.2196/23019

**Published:** 2020-11-18

**Authors:** Abrar Al-Hasan, Jiban Khuntia, Dobin Yim

**Affiliations:** 1 Kuwait University AlShadadiya University City Kuwait; 2 University of Colorado Denver Denver, CO United States; 3 Loyola University Maryland Baltimore, MD United States

**Keywords:** COVID-19, adherence, coping appraisal, threat appraisal, protection motivation theory, social distancing, information sources, social media, knowledge, coping, threat, protection, motivation, cross-sectional, survey

## Abstract

**Background:**

Social distancing is an effective preventative policy for COVID-19 that is enforced by governments worldwide. However, significant variations are observed in adherence to social distancing across individuals and countries. Due to the lack of treatment, rapid spread, and prevalence of COVID-19, panic and fear associated with the disease causes great stress. Subsequent effects will be a variation around the coping and mitigation strategies for different individuals following different paths to manage the situation.

**Objective:**

This study aims to explore how threat and coping appraisal processes work as mechanisms between information and citizens’ adherence to COVID-19–related recommendations (ie, how the information sources and social media influence threat and coping appraisal processes with COVID-19 and how the threat and coping appraisal processes influence adherence to policy guidelines). In addition, this study aims to explore how citizens in three different countries (the United States, Kuwait, and South Korea), randomly sampled, are effectively using the mechanisms.

**Methods:**

Randomly sampled online survey data collected by a global firm in May 2020 from 162 citizens of the United States, 185 of Kuwait, and 71 of South Korea were analyzed, resulting in a total sample size of 418. A seemingly unrelated regression model, controlling for several counterfactuals, was used for analysis. The study’s focal estimated effects were compared across the three countries using the weighted distance between the parameter estimates.

**Results:**

The seemingly unrelated regression model estimation results suggested that, overall, the intensity of information source use for the COVID-19 pandemic positively influenced the threat appraisal for the disease (*P*<.001). Furthermore, the intensity of social media use for the COVID-19 pandemic positively influenced the coping appraisal for the disease (*P*<.001). Higher COVID-19 threat appraisal had a positive effect on social distancing adherence (*P*<.001). Higher COVID-19 coping appraisal had a positive effect on social distancing adherence (*P*<.001). Higher intensity of COVID-19 knowledge positively influenced social distancing adherence (*P*<.001). There were country-level variations. Broadly, we found that the United States had better results than South Korea and Kuwait in leveraging the information to threat and coping appraisal to the adherence process, indicating that individuals in countries like the United States and South Korea may be more pragmatic to appraise the situation before making any decisions.

**Conclusions:**

This study’s findings suggest that the mediation of threat and coping strategies are essential, in varying effects, to shape the information and social media strategies for adherence outcomes. Accordingly, coordinating public service announcements along with information source outlets such as mainstream media (eg, TV and newspaper) as well as social media (eg, Facebook and Twitter) to inform citizens and, at the same time, deliver balanced messages about the threat and coping appraisal is critical in implementing a staggered social distancing and sheltering strategy.

## Introduction

COVID-19 has instilled fear among all individuals across the world. Since beginning in Wuhan, China in December 2019, the COVID-19 pandemic has led to more than 6.5 million cases and 390,000 deaths reported worldwide as of June 4, 2020 [1].

Although there is no treatment or vaccine for COVID-19 [2] yet, the mitigation approach to COVID-19 has focused on infection control, effective quarantine, and treatment cure rates [3,4]. Infected individuals do not often exhibit any symptoms, and the disease progresses swiftly and kills patients at a much higher rate than the typical flu [5]. Limited testing availability, combined with few treatment options other than a ventilator to assist breathing, forces individuals to adopt a varying level of preventive measures [6-9], including measures to help alleviate psychological impacts [10,11].

Individuals can practice good respiratory hygiene by washing hands with soap and water often for at least 20 seconds and avoiding touching the eyes, nose, or mouth with unwashed hands. More drastic efforts include social distancing or maintaining a safe distance from others and sheltering practices by staying at home to avoid all contacts [2].

There are variations in citizens’ adherence to the COVID-19–related social distancing guidelines suggested by governments across countries to manage and mitigate the disease, aligning to the preventive measures [12], for example, whether or not to wear a mask [13,14]. Citizen’s willingness to follow the guidance will depend on the fear and anxiety caused by the disease. Arguably, compared to other diseases, infectious diseases like COVID-19 induce fear because of known and unknown reasons associated with spread and prevalence, the high rates of morbidity and mortality, and subsequent societal stigma and discrimination associated with the diagnosis and treatment of the disease [15-18].

Along with the actual nature and impact of COVID-19, the information available through different channels and the discussions through social media have played a significant role in influencing people’s mindsets. The COVID-19 pandemic is associated with sleep disturbance and suicidal thoughts [19], misinformation and distress [20,21], and greater self-confidence when receiving information from more sources but only for health care workers [22]. Thus, the culmination of information and social media influences can influence the fear and coping strategies related to the disease and subsequent adherence to the policy recommendations [23].

The panic and fear associated with the disease will create a struggle in people’s mindsets, including creating worst-case scenarios around their situations [24]. Subsequent effects will be a variation around the coping and mitigation strategies for different individuals following different paths to manage the situation.

In this context, this study asks two research questions: (1) How do the information sources and social media influence the threat and coping appraisal process with COVID-19? and (2) How does the threat and coping appraisal process influences adherence to policy guidelines?

The COVID-19 situation poses a sense of helplessness in that there is no treatment, and thus, individuals rely on different information sources to learn about the disease. Because they cannot see a doctor as a preassessment or plan for the disease, the information gained from the press, television, and internet influences their disease management and mitigation strategy. Thus, individual behavior would be affected by these information sources, as individuals will try to understand and maneuver complex situations [25-28]. Furthermore, given that social media is emerging as a critical information source to influence individual beliefs and perceptions, it is beyond doubt that such an influence would be quite useful in the COVID-19 situation [29-31].

Threat and coping appraisal mechanisms, grounded in the protection motivation theory (PMT), have been suggested to be precursors of individuals’ actions to protect against infectious diseases [32] such as the influenza outbreak [33]. The intention to adopt a protective behavior such as adhering to the social distance recommendations results from perceiving a given threat such as a disease and desiring to avoid the adverse outcomes of such a threat [34]. The protection motivation perspective suggests that health risk is appraised by considering what the threat is because of the severity of a disease or health issue (threat appraisal: severity), how vulnerable an individual perceives the disease or health issue (threat appraisal: vulnerability), how successful preventative behavior is (coping appraisal: response efficacy), and how confident the individual feels in preventing the risk (coping appraisal: self-efficacy). Although several extensions and applications of the protection motivation concept are applied to health contexts, the basic premise of threat appraisal, consisting of severity and vulnerability, and coping appraisal, consisting of self and response efficacies, have remained similar (for a meta-analysis, see [34,35]).

Taking a closer look at people’s actions, thoughts, or emotions can be a complicated process. However, resisting or suppressing our emotions creates paradoxical outcomes such as aggravating our fears instead of making them vanish. Thus, these coping strategies embrace and approach our stressors head-on to build grit and resilience. It is normal to find it challenging to be consistent when starting a new routine. In this context, exploration of COVID-19 mitigation using threat and coping appraisal mechanisms is essential.

Based on these discussions, this study focuses explicitly on testing the following hypotheses:

1. The intensity of the use of information sources for COVID-19 positively influences the threat appraisal for the disease.
2. The intensity of social media use for COVID-19 positively influences the coping appraisal for the disease.
3. A higher COVID-19 threat appraisal positively influences citizens’ adherence intentions.
4. A higher COVID-19 coping appraisal positively influences citizens’ adherence intentions.

This study conducts a comparative evaluation of the citizens’ adherence process to COVID-19–related recommendations by the governments in three different countries: the United States, Kuwait, and South Korea.

## Methods

### Recruitment

This study started with a discussion in a focus group in Kuwait. The ten people who participated in the focus group opined that assessing threat and coping strategies is essential to manage the COVID-19 situation. Along with this insight, the group also suggested that different cultural systems and relevant mindsets will differ in adhering to government recommendations. This motivated us to study the research question across different countries with polarized mindsets and different cultural systems. Thus, the study expanded to other countries with different cultures. Due to resource constraints, the sampling was limited to countries that the authors have firsthand experience in explaining the similarities and differences.

A global survey-deploying firm collected the data for this study using online platforms. The firm recruited respondents from the United States, Kuwait, and South Korea in May 2020. The firm sampled respondents using an age, gender, ethnicity, and geographic region–based strata and quota matching process. Participation in the survey was free and voluntary; the respondents filled in electronic informed consent that was shown on the first page of the survey. The firm protects the confidentiality of anonymous respondents.

### Data Collection

Data was collected using a survey instrument, as shown in Multimedia Appendix 1 Table A1. The questions asked participants about the cause and current state of the COVID-19 situation, their opinion on the government’s role during the COVID-19 pandemic, the use of health information sources and social media for COVID-19–related information, and PMT measures for the COVID-19 pandemic adapted from previously validated scales [36-39]. The survey items included simple information-seeking questions and several existing validated scales from prior studies [35,40-47].

The survey instrument was pilot-tested using a sample of 48 respondents, leading to minor refinements to a few items. A total of 482 participants took the survey. Because of missing responses to the items, 64 observations were excluded, resulting in a sample size of 418. Responses were coded, validated, and analyzed using Stata version 16 (StataCorp).

### Sample Demographics

Table 1 shows the descriptive statistics and pairwise correlations among the key variables used in this study. Out of 418 participants, 299 (58.7%) were female. The sample’s largest age group was 18-27 years (n=192, 37.3%). This group was followed by the 28-37 years age group (n=150, 29.1%), 38-47 years age group (n=70, 13.6%), 48-57 years age group (n=43, 8.4%), and 58 years or older group (n=60, 11.7%). Multimedia Appendix 1 Figure A1 shows the countrywise comparison of the respondents’ age. In terms of income level, 102 (20.5%) participants make less than US $30,000 annually; 92 (18.5%) make US $30,000-US $50,000; 102 (20.5%) make US $50,000-US $80,000; 65 (13.1%) make US $80,000-US $100,000; 57 (11.5%) make US $100,000-US $150,000; and 79 (15.9%) make more than US $150,000. The household income distribution as varied by country is shown in Multimedia Appendix 1 Figure A2. A detailed distribution of several demographic controls used in the models is available in Multimedia Appendix 1 Table A2.

**Table 1:** Summary statistics and pairwise correlations among key variables (N=418).

Variabless	Mean (SD)	Min	Max	1	2	3	4	5	6	7	8	9	10	11	12	13
Adherence (1)	4.18 (1.00)	1	5	1.00												
Threat appraisal (2)	3.32 (0.87)	1	5	0.38	1.00											
Severity (3)	3.55 (0.97)	1	5	0.45	0.87	1.00										
Vulnerability (4)	3.10 (1.01)	1	5	0.21	0.89	0.55	1.00									
Coping appraisal (5)	2.93 (0.78)	1	5	0.53	0.44	0.46	0.32	1.00								
Self-efficacy (6)	3.06 (1.51)	1	5	0.28	0.31	0.30	0.25	0.84	1.00							
Response efficacy (7)	4.29 (0.89)	1	5	0.64	0.42	0.46	0.27	0.77	0.30	1.00						
Knowledge (8)	15.04 (3.62)	4	24	0.33	0.10	0.18	0.01	0.33	0.17	0.39	1.00					
Information source (9)	2.26 (1.25)	0	5	0.16	0.08	0.08	0.05	0.17	0.04	0.27	0.19	1.00				
Social media (10)	1.65 (1.43)	0	7	0.03	0.17	0.17	0.12	0.08	0.13	0.00	0.02	0.40	1.00			
Age (11)	2.27 (1.35)	1	5	0.00	0.00	0.03	–0.03	0.00	–0.10	0.12	0.09	0.21	0.04	1.00		
Female (12)	0.59 (0.49)	0	1	0.11	0.11	0.09	0.11	0.15	0.17	0.07	0.13	0.09	0.08	0.00	1.00	
Household income (13)	2.24 (1.72)	0	5	0.05	–0.07	–0.03	–0.09	0.13	0.14	0.06	0.11	0.04	–0.04	0.04	–0.12	1.00

### Study Variables

The main dependent variable in this study is *adherence*. As shown in Multimedia Appendix 1 Table A1, *adherence* was measured using three questions of whether they would comply with the social distancing measures. The items’ internal consistencies were tested using Cronbach alpha (0.81), and the standardized score was generated for the *adherence* variable. Table 1 shows that, on average, *adherence* is 4.18 out of 5, showing that most people are adhering to social distancing recommendations. The mean adherence level in Kuwait was the highest (mean 4.53, SD 0.81), followed by the United States (mean 4.14, SD 0.92) and South Korea (mean 3.53, SD 1.18). Multimedia Appendix 1 Figure A3 displays the mean adherence across countries.

Three main independent variables were of interest in this study to examine *adherence*. First, the independent variable *threat appraisal* consists of both the severity and vulnerability of the situation [46]. *Severity* is the perceived degree of harm from engaging in unhealthy behavior, the extent to which one will experience or die from contracting COVID-19 upon not following social distancing recommendations. *Vulnerability* is the perceived probability of threat occurrence, the extent to which one will contract COVID-19 upon not following social distancing recommendations. Both variables were operationalized using three questions adopted from previous studies to assess the *severity* and *vulnerability* of the COVID-19 pandemic (see Multimedia Appendix 1 Table A1). The items’ internal consistency was tested using Cronbach alpha (0.63 and 0.76, respectively), and the standardized score was generated for both *severity* and *vulnerability* variables.

The second main independent variable was *coping appraisal*, which consists of both self-efficacy and response efficacy of the situation [46]. *Self-efficacy* is the perceived belief that one can successfully maintain a safe distance from others when in contact or stay home to avoid all contacts. *Response efficacy* is the perceived efficacy of adherence that adopting social distancing will be effective in reducing the threat of COVID-19. Both variables were operationalized using three questions adopted from previous studies to assess the *self-efficacy* and *response efficacy* of adherence to the COVID-19 pandemic regulations (see Multimedia Appendix 1 Table A1). The items’ internal consistency was tested using Cronbach alpha (0.6 and 0.8, respectively), and the standardized score was generated for both *self-efficacy* and *response efficacy* variables.

Lastly, the *knowledge* variable was coded to reflect the respondents’ overall knowledge of COVID-19, as displayed in Multimedia Appendix 1 Table A1. The mean for *knowledge* was 15.04, with a minimum of 4 and a maximum of 24. The mean COVID-19 *knowledge* was highest in Kuwait (mean 16.32, SD 3.05), followed by the United States (mean 15.04, SD 3.62) and South Korea (mean 11.85, SD 3.74). Multimedia Appendix 1 Figure A4 displays the mean knowledge across countries.

To examine *threat appraisal* and *coping appraisal*, this study focuses on *COVID-19 information sources* used and on *COVID-19 social media* use. *COVID-19 information sources* was operationalized as the total number of sources used to attain COVID-19 health information. Table 1 displays the mean for the whole sample as 2.26, showing that individuals use two information sources on average to attain COVID-19 information. The mean was highest in Kuwait (mean 2.50, SD 1.13), followed by the United States (mean 2.31, SD 1.34), and South Korea (mean 1.68, SD 1.11). *COVID-19 social media* was also operationalized as the total number of social media platforms used to attain COVID-19 health information. Table 1 displays the mean for the whole sample as 1.16, showing that individuals use one social media platform to attain COVID-19 information. The mean was highest in Kuwait (mean 2.10, SD 1.38), followed by South Korea (mean 1.48, SD 1.36) and the United States (mean 1.29, SD 1.41). *COVID-19 social media* was further examined by categorizing the platforms to social network platforms (Facebook and LinkedIn), media sharing platforms (Instagram, Snapchat, TikTok, and YouTube), and texting and microblogging platforms (Twitter and Whatsapp). Details of the analysis are shown in Multimedia Appendix 1 Table A5.

In addition to these key variables of interest, several control variables such as age, gender, and household income were included to account for counterfactual explanations relevant to our models (see Multimedia Appendix 1 Table A1 for details).

### Econometric Analysis

Following PMT, the empirical model specifies how individuals express their opinion toward *adherence* of social distancing guidance by the government through *threat appraisal*, *coping appraisal*, and *knowledge* of COVID-19. Furthermore, *threat appraisal* and *coping appraisal* were specified through COVID-19 sources of information (*COVID-19 information sources* and *COVID-19 social media*). A set of control variables to enhance our empirical model’s robustness included demographics characteristics of the survey participants, such as *gender*, *age*, and *household income*. The formal specification of the general model is as follows:

Threat appraisal model:

*Threat appraisal_i_ = β_0_ + β_1_ × COVID-19 information sources_i_ + β_2_ × COVID-19 social media_i_ + β_3_ × Country + Controls_i_ +* ɛ*i*   **(1)**

Coping appraisal model:

*Coping appraisal_i_ = β_0_ + β_1_ × COVID-19 information sources_i_ + β_2_ × COVID-19 social media_i_ + β_3_ × Country + Controls_i_ +* ɛ*i*   **(2)**

Adherence model:

*Adherence_i_ = β_0_ + β_1_ × Threat appraisal_hat_i_ + β_2_ × Coping appraisal_hat_i_ + β_3_ × Knowledge_i_ + β_4_ × Country + Controls_i_ +* ɛ*i*   **(3)**

Where *controls* includes *gender*, *age groups*, and *household income*. The *country* dummy was included in the full sample model but was removed for subsample analyses.

Seemingly unrelated regression (SUR) was used to estimate the *β* coefficients of the key parameters and employ robust standard errors to test the models. are the disturbances associated with each observation. SUR was used to estimate to what extent our set of key variables influence *adherence*. The adherence model (Equation 3) uses the predicted values of threat appraisal (*threat appraisal_hat*) and coping appraisal (*coping appraisal_hat*) from the first stage models (Equations 1 and 2).

## Results

Table 2 presents the key estimation results for Equations 1-3 for the whole sample. The first column (1) in the table shows the parameter estimates for the *coping appraisal* dependent variable, column (2) shows the parameter estimates for the *threat appraisal* dependent variable, and column (3) shows the parameter estimates for the *adherence* dependent variable for the full sample. Table 3 displays the key estimation results for Equations 1 and 2 for the individual countries. Columns 1-3 show the parameter estimates for the *coping appraisal* dependent variable, and columns 4-6 display the parameter estimates for the *threat appraisal* dependent variable for the United States, South Korea, and Kuwait, respectively. Table 4 presents the key estimation results for the *adherence* dependent variable, as in Equation 3, for the United States, South Korea, and Kuwait. Multimedia Appendix 1 Table A3 further analyzes the *adherence* model using the constituent variables (*severity*, *vulnerability*, *self-efficacy*, and *response efficacy*).

**Table 2:** Seemingly unrelated regression model results for the full sample.

Variables	DV^a^: coping appraisal (1)		DV: threat appraisal (2)		DV: adherence (3)	
	Full sample	*P* value	Full sample	*P* value	Full sample	*P* value
COVID-19 information source	0.112 (0.03)^b^	<.001	0.034 (0.04)	.35	N/A^c^	N/A
COVID-19 social media	0.034 (0.03)	.35	0.112 (0.03)	<.001	N/A	N/A
Threat appraisal	N/A	N/A	N/A	N/A	0.215 (0.05)	<.001
Coping appraisal	N/A	N/A	N/A	N/A	0.511 (0.06)	<.001
Knowledge	N/A	N/A	N/A	N/A	0.061 (0.01)	<.001
Age	–0.008 (0.03)	.72	0.011 (0.03)	.72	0.006 (0.03)	.85
Female	0.213 (0.08)	.04	0.174 (0.09)	.04	0.038 (0.08)	.64
Household income	0.055 (0.02)	.15	–0.037 (0.03)	.15	–0.002 (0.02)	.94
Constant	2.328 (0.13)	<.001	2.936 (0.15)	<.001	1.034 (0.23)	<.001
Observations, n	418	N/A	418	N/A	418	N/A
*R* ^2^	0.076	N/A	0.051	N/A	0.375	N/A
Chi-square	34.25	N/A	22.37	N/A	249.90	N/A
Root mean square error	0.7482	N/A	0.8420	N/A	N/A	N/A
*P* value	N/A	<.001	N/A	<.001	N/A	<.001

^a^DV: dependent variable.

^b^Standard errors in parentheses.

^c^N/A: not applicable.

**Table 3:** Coping and threat appraisal seemingly unrelated regression model results for individual countries.

Variables	DV^a^: coping appraisal							DV: threat appraisal					
	US (1)	*P* value	South Korea (2)	*P* value	Kuwait (3)	*P* value	US (4)		*P* value	South Korea (5)	*P* value	Kuwait (6)	*P* value
COVID-19 information source	0.099 (0.04)^b^	.01	0.221 (0.08)	.007	0.053 (0.04)	N/A^c^	0.021 (0.05)		.68	0.213 (0.12)	.07	0.077 (0.05)	.11
COVID-19 social media	–0.007 (0.04)	.86	–0.029 (0.08)	.71	–0.009 (0.03)	N/A	0.173 (0.05)		<.001	–0.122 (0.11)	.28	0.011 (0.04)	.77
Age	–0.0908 (0.04)	.01	–0.002 (0.08)	.98	0.045 (0.03)	N/A	–0.043 (0.05)		.35	0.040 (0.11)	.72	0.052 (0.04)	.19
Female	0.032 (0.11)	.76	0.058 (0.18)	.75	0.095 (0.08)	N/A	0.065 (0.14)		.63	–0.122 (0.26)	.63	0.246 (0.10)	.02
Household income	–0.030 (0.03)	.34	0.015 (0.07)	.82	0.029 (0.02)	N/A	–0.0917 (0.04)		.02	0.036 (0.10)	.71	–0.055 (0.03)	.06
Constant	2.652 (0.18)	<.001	1.726 (0.29)	<.001	3.042 (0.14)	N/A	2.907 (0.23)		<.001	2.883 (0.42)	<.001	3.212 (0.18)	<.001
Observations, n	162	N/A	71	N/A	185	N/A	162		N/A	71	N/A	185	N/A
*R* ^2^	0.075	N/A	0.117	N/A	0.048	N/A	0.139		N/A	0.050	N/A	0.063	N/A
Chi-square	13.20	N/A	9.47	N/A	9.27	N/A	26.18		N/A	3.77	N/A	12.43	N/A
Root mean square error	0.6307	N/A	0.6834	N/A	0.4996	N/A	0.8166		N/A	0.9817	N/A	0.6581	N/A
*P* value	N/A	.02	N/A	.09	N/A	.10	N/A		<.001	N/A	.58	N/A	.03

^a^DV: dependent variable.

^b^Standard errors in parentheses.

^c^N/A: not applicable.

**Table 4:** Adherence seemingly unrelated regression model results for individual countries.

Variables	DV^a^: adherence					
	US (1)	*P* value	South Korea (2)	*P* value	Kuwait (3)	*P* value
Threat appraisal	0.223 (0.08)^b^	.006	0.576 (0.12)	<.001	0.0504 (0.08)	.55
Coping appraisal	0.334 (0.11)	.002	0.531 (0.16)	.001	0.536 (0.11)	<.001
Knowledge	0.053 (0.02)	.01	0.047 (0.03)	.07	0.040 (0.02)	.03
Age	0.038 (0.05)	.42	–0.012 (0.08)	.88	–0.038 (0.04)	.39
Female	–0.025 (0.14)	.86	0.202 (0.17)	.24	0.043 (0.12)	.72
Household income	0.013 (0.04)	.77	–0.044 (0.06)	.49	–0.011 (0.03)	.74
Constant	1.644 (0.45)	<.001	–0.135 (0.39)	.73	1.968 (0.52)	<.001
Observations, n	162	N/A^c^	71	N/A	185	N/A
*R* ^2^	0.180	N/A	0.669	N/A	0.159	N/A
Chi-square	35.16	N/A	146.67	N/A	33.65	N/A
Root mean square error	0.8481	N/A	0.6629	N/A	0.7399	N/A
*P* value	N/A	<.001	N/A	<.001	N/A	<.001

^a^DV: dependent variable.

^b^Standard errors in parentheses.

^c^N/A: not applicable.

The estimated coefficients were further compared across countries using a model-based chi-square comparison test with Bonferroni adjustments, as shown in Table 5. Multimedia Appendix 1 Table A4 further compares the coefficients across countries on the detailed PMT variables (*severity*, *vulnerability*, *self-efficacy*, and *response efficacy*).

**Table 5:** Comparison of coefficients across countries on the main variables.

Variables		US vs Kuwait		US vs South Korea		South Korea vs Kuwait	
		Chi-square^a^	*P* value	Chi-square	*P* value	Chi-square	*P* value
**DV^b^: threat appraisal**							
	COVID-19 information sources	0.49	.48	3.19	.07	1.98	.16
	COVID-19 social media	5.59	.02	4.98	.03	1.17	.28
**DV: coping appraisal**							
	COVID-19 information sources	0.38	.54	2.33	.13	3.96	.047
	COVID-19 social media	0.03	.87	0.02	.88	0.06	.80
**DV: adherence**							
	Threat appraisal	0.27	.49	11.91	<.001	12.45	<.001
	Coping appraisal	4.47	.049	1.66	.27	0.25	.62
	Knowledge	0.24	.72	0.04	.81	0.01	.94

^a^Chi square values reported with Bonferroni adjustment.

^b^DV: dependent variable.

The first set of findings examines the *adherence* model in Tables 2 and 4. We first examined the effect of the *threat appraisal* variable on *adherence*. As shown in Tables 2 and 4, coefficients of *threat appraisal* were positive and statistically significant at *P*<.001 across the full sample (Table 2 column 3), the United States (*P*=.006; Table 4 column 1), and South Korea (*P*<.001; Table 4 column 2). This suggests that individuals who are threatened by the COVID-19 pandemic positively follow adherence guidance on social distancing. Further analysis of the *severity* coefficients in Multimedia Appendix 1 Table A3 shows positive and statistically significant influence on adherence (column 1: all sample *P*<.001; column 2: the United States *P*=.03; column 3: South Korea *P*<.001; column 4: Kuwait *P*<.001). However, as shown in Multimedia Appendix 1 Table A3, *vulnerability* was not statistically significant in any of the samples. This finding shows that *threat appraisal* was mainly led by *severity* in terms of *adherence* to COVID-19 social distancing regulations. As displayed in Table 5, comparing the coefficients across countries, we found that the significant positive effect of *threat appraisal* on *adherence* shows that individuals who reside in the United States are more likely to follow adherence than South Korea, and those that reside in South Korea are more likely to follow adherence than those in Kuwait (*P*<.001).

Similarly, as displayed in Multimedia Appendix 1 Table A4, comparing the coefficients across countries, we found that the significant positive effect of *severity* on *adherence* shows that individuals who reside in the United States are more likely to follow adherence than South Korea (*P*=.02), and those in South Korea are more likely to follow adherence than those in Kuwait (*P*=.046). As shown in Multimedia Appendix 1 Table A4, in terms of *vulnerability*, the positive and significant effect showed that *vulnerability* was more influential on *adherence* for individuals that reside in South Korea than Kuwait (*P*=.07).

We subsequently examined the effect of *coping appraisal* on *adherence*. As shown in Tables 2 and 4, we found that the coefficient of *coping appraisal* was positive and statistically significant for all the models (Table 2 column 1: full sample, *P*<.001; Table 4 column 1: the United States, *P*=.002; Table 4 column 2: South Korea, *P*=.001; Table 4 column 3: Kuwait, *P*<.001). Further analysis in Multimedia Appendix 1 Table A3 showed that *self-efficacy* was positive and statistically significant in the full model (*P*=.02) and Kuwait only (*P*=.01). However, *response efficacy* was positive and statistically significant in all the models (column 1: full sample, *P*<.001; column 2: the United States, *P*<.001; column 3: South Korea, *P*<.001; column 4: Kuwait, *P*=.001). Interestingly, across countries, we found that, as displayed in Table 5, the significant positive impact of *coping appraisal* showed that those in the United States were more likely to follow adherence than those in Kuwait (*P*=.049). In terms of *self-efficacy* across countries, as shown in Multimedia Appendix 1 Table A4, we found the significant positive effect of *self-efficacy* on *adherence* showed that individuals who reside in the United States and South Korea are more likely to follow adherence than those in Kuwait (*P*<.001 and *P*=.04, respectively). As for *response efficacy* across countries, there was no significant comparative difference in this effect, as the comparative chi-square values were not significant, as shown in Multimedia Appendix 1 Table A4.

We then examined the effect of *knowledge* on *adherence*. As shown in Tables 2 and 4, the coefficients of *knowledge* were positive and statistically significant (Table 2 column 3: all sample, *P*<.001; Table 4 column 1: the United States, *P*=.01; Table 4 column 3: Kuwait *P*=.03) on *adherence* except for in South Korea. As shown in Table 5, comparisons of coefficients for *knowledge* across the three countries did not show statistically significant results.

The second set of findings examined the *threat appraisal* model in Tables 2 and 3. As shown in Table 2 column 2, *COVID-19 social media* coefficients were positive and statistically significant at *P*<.001 across the full sample and the United States (Table 3 column 4). This suggests that social media platforms have an impact on threat appraisal. However, we found no significance for *COVID-19 information sources*. Comparing coefficients across countries as shown in Table 5, we found that the significant positive effect of *COVID-19 social media* on *threat appraisal* showed that individuals who reside in the United States were more likely to have higher *threat appraisal* than those in Kuwait (*P*=.02) and those in South Korea (*P*=.03).

Further analyzing the social media platform categories as shown in Multimedia Appendix 1 Table A5, in the United States, social network platforms such as Facebook and LinkedIn positively influenced threat appraisal (*P*=.03), and text-based or microblogging platforms such as Twitter and Whatsapp positively influenced threat appraisal in the whole sample (*P*<.001) and in the United States (*P*=.007). Some controls of the *threat appraisal* model in Tables 2 and 3 were significant. Females displayed higher *threat appraisal* than males in the full sample and in Kuwait (Table 2 column 2: *P*=.04 and Table 3 column 6: *P=*.02, respectively). In the United States, the lower the household income, the higher the *threat appraisal* (Table 3 column 4: *P*=.02).

The last set of findings examined the *coping appraisal* model in Tables 2 and 3. In contrast to the *threat appraisal* model, *COVID-19 social media* displayed no significance. However, coefficients for *COVID-19 information sources* were positive and significant for the whole sample (Table 2 column 1: *P*<.001), the United States (Table 3 column 1: *P*=.01), and South Korea (Table 3 column 2: *P*=.007). This finding suggests that using more information sources has an impact on the coping appraisal. Comparing coefficients across countries as shown in Table 5, we found that the significant positive effect of *COVID-19 information sources* on *coping appraisal* showed that individuals who reside in South Korea were more likely to have higher *coping appraisal* than those in Kuwait (*P*=.047).

Further analyzing the social media platform categories as shown in Multimedia Appendix 1 Table A5, social network platforms such as Facebook and LinkedIn negatively influenced coping appraisal on the whole sample (*P=*.001), and text-based or microblogging platforms such as Twitter and Whatsapp positively influenced coping appraisal in the whole sample (*P*<.001). Some controls of the *coping appraisal* model in Tables 2 and 3 were significant. Older individuals in the United States had lower *coping appraisal* (Table 3 column 1: *P*=.01). Compared to males, females had higher *coping appraisal* in the full model (Table 2 column 1: *P*=.005). Those with higher household income had higher *coping appraisal* in the full model (Table 2 column 1: *P=*.01).

## Discussion

### Principal Findings

In general, this study found that coping appraisal, threat appraisal, and knowledge positively influence adherence. Furthermore, using various COVID-19 information sources influences coping appraisal, and using social media for COVID-19 information influences threat appraisal. Tables 6 and 7 summarize the findings from this study (Multimedia Appendix 1 Table A6 displays the summary of findings on the specific PMT constructs and the social media platform constructs). In this section, we elaborate on the findings to provide useful policy and managerial insights.

**Table 6:** Summary of findings (part 1).

Variables	Coping appraisal				Threat appraisal				Findings
	All	US	South Korea	Kuwait	All	US	South Korea	Kuwait	
COVID-19 information sources	Pos^a^	Pos	Pos	NS^b^	NS	NS	NS	NS	H1^c^: Partially supported (supported for the whole sample, US, and South Korea). Using more information sources to get COVID-19 information positively influences coping appraisal. Information sources are more influential on coping appraisals in South Korea than Kuwait. No comparative difference in threat appraisal across countries.
COVID-19 social media	NS	NS	NS	NS	Pos	Pos	NS	NS	H2: Partially supported (supported for the whole sample and US). Using social media to get COVID-19 information positively influences threat appraisal. Social media is more influential on threat appraisal in US than Kuwait and South Korea. No comparative difference in coping appraisal across countries.

^a^Pos: positive association.

^b^NS: not significant.

^c^H: hypothesis.

**Table 7:** Summary of findings (part 2).

Variables	Adherence				Findings	
	All	US	South Korea	Kuwait		
Threat appraisal	Pos^a^	Pos	Pos	NS^b^		H3^c^: Partially supported (supported for the whole sample, US, and South Korea). Threat appraisal positively influences social distancing adherence. Threat appraisal is more influential in US than in South Korea, and more in South Korea than in Kuwait.
Coping appraisal	Pos	Pos	Pos	Pos		H4: Supported. Coping appraisal positively influences social distancing adherence. Coping is more influential in US than Kuwait in terms of adherence.
Knowledge	Pos	Pos	NS	Pos		Knowledge positively influences social distancing adherence in the whole sample, US, and Kuwait. No comparative difference in results across countries.

^a^Pos: positive association.

^b^NS: not significant.

^c^H: hypothesis.

First, the study found that using more information sources positively influences the coping appraisal associated with the COVID-19 pandemic situation. Undoubtedly, this finding’s importance relates to the increasing use and trade-off of information sources by the public. As much as people like to use many information sources, whether it is beneficial or not remains a question. Furthermore, citizens may be swayed by popular information sources such as the internet, TV, and newspapers. This finding highlights the benefits of using multiple information sources during the COVID-19 pandemic. This could be due to the high amount of misinformation and that the pandemic is ever-evolving. Therefore, attaining multiple information sources allows individuals to obtain more accurate information that helps with their coping appraisal. This finding is in line with a recent COVID-19 study that displayed an association between more sources of information and higher self-confidence to cope with COVID-19 in health care workers, yet the direction of the association was not confirmed in their study [22]. Thus, this study sheds light on this literature and displays the importance of multiple sources of information to cope with the COVID-19 pandemic [48]. The study also found that information sources are more influential on the coping appraisal in South Korea than Kuwait, displaying that individuals in South Korea are more rationally using information sources to cope than individuals in Kuwait.

Second, this study found that using social media to get COVID-19 information positively influences threat appraisal. This is an important finding to validate the increasing trustworthiness of social media on people’s decisions and the role of social media in this pandemic. Social media is one of the main channels used to provide updated COVID-19 information [49]. This study’s findings are consistent with previous studies [50] that showed that the frequency of social media was associated with high odds of anxiety [51]. This association might be because, throughout the COVID-19 pandemic, many false reports and misinformation bombarded social media, which resulted in a lot of confusion and anxiety [50]. Furthermore, many individuals use social media to express their feelings of worry, anxiety, nervousness, and fear, which is contagious in social networks [52]. As we stated earlier, the social media role in people’s minds during the pandemic is not free from harmful aspects such as increased anxiety, sleep disturbance, suicidal thoughts[19], misinformation, and distress [20,21]. Thus, it is interesting to note the positive aspects such as social media’s role in adherence decisions. As for country comparisons, we found that social media is more influential on threat appraisal in the United States than in South Korea and Kuwait. A possible reason for this finding is that social media information in the United States might be more fearful or driven with more riddles (ie, whether to trust Twitter postings or not) compared to South Korea and Kuwait.

Third, threat appraisal positively influences social distancing adherence. One of the primary emotional responses during a pandemic is fear [53]. This fear is a defensive system to combat ecological threats [54]. A meta-analysis found that targeting fears can be useful in situations, such that appealing to fear leads people to change their behavior if they feel capable of dealing with the threat but leads to defensive reactions when they feel helpless to act [55]. This study sheds light on this stream of research by displaying that fear or threat appraisal was useful in the COVID-19 pandemic since it changes an individual’s behavior and influences their social distancing adherence.

Based on PMT, a fear appeal is a cognitive assessment that prepares individuals against the severity of a threatening event. People consider the pros and cons, and the probability of the event occurring to develop a response. The recommended response in the COVID-19 context is adherence. The cognitive assessment enhances the fear appeal and subsequent motivation to protect oneself—without which the recommended action would not be effective across citizens. Besides, there is a more substantial threat appraisal for social distance adherence in the United States than in South Korea and Kuwait. This follows through with the social media country comparison finding; in addition, the finding shows that threat appraisal in the United States is more rational than that of South Korea and Kuwait.

Fourth, coping appraisal positively influences social distancing adherence, and this effect is more influential in the United States than Kuwait, again displaying that individuals in the United States are more pragmatic in the coping and adherence process. One of the main factors that have been stressed upon during the COVID-19 pandemic is how to cope with the current situation [15]. Studies have shown that problem-focused coping is associated with better adherence to health-related behaviors and a higher sense of control [56,57]. The findings support these perspectives and suggest, as per PMT, that the preventive actions will be preferred in a high threat situation when both the self-efficacy and the efficacy of the adherence plans are high. This finding agrees with this research stream and displays the importance of coping appraisal during the COVID-19 pandemic to adhere to social distancing recommendations.

These findings partially explain the variations observed in the adherence outcomes, which can be explained due to the variations observed in the threat and coping appraisals. Finally, as an additional outcome, we found that knowledge positively influences social distancing adherence in the whole sample, the United States, and Kuwait. There were no comparative difference in results across countries. This sheds light on the importance of correct knowledge during this pandemic to be able to adhere to social distancing recommendations. Association between adherence and better knowledge of the disease is consistent with recent findings and provides evidence for using proper interventions of proper communication related to the disease on improving adherence [56,58].

### Practice and Policy Implications

A set of implications and recommendations for public health officials and policy makers can be drawn from this study. This study points to the gap in the responsible behavior of individuals regarding adherence. The current government efforts to mitigate and the expectation that everything should be back to normal have differing consequences. Existing work suggests that policy recommendations’ efficacy and outcomes depend on the individuals’ beliefs and subsequent actions [59,60]. The first step in this process is the firm belief of whether the recommended action will mitigate the threat or manage the fearful situation.

Thus, the key to minimizing rejection and maximizing acceptance of recommendation is reliant on the messages and information provided to citizens. Often, the media outlets and information sources are left to craft their messages independently or freely without the policy makers’ recommended guidelines. There needs to be policy guidelines with careful attention about what messages to give and how to give them, so that it does not instill too much fear nor allow citizens to be too careless (ie, messages and information need to have a balance to involve appropriate threat and coping appraisals in citizens’ minds).

### Limitations

This study examines factors that influence citizens adhering to social distancing at a point in time. However, the citizen might go back and forth in the adherence process, and the threat appraisal, coping appraisal, knowledge level, and sources of information may change over time. This is a limitation of this study, as the data set used is a cross-sectional survey. Future studies could examine how a citizen’s coping appraisal, threat appraisal, and adherence changes over time.

In addition, using the random sampling process in the United States, Kuwait, and South Korea samples may include fewer familiar respondents to the study context. In particular, the questionnaire was only online. Therefore, respondents were all users of the internet. The study does not examine noninternet users, which could have differential impacts. Thus, the generalization of the sample to a uniform national culture characteristic is a limitation of this study. Future studies could conduct online and offline surveys, and examine the difference in threat and coping appraisal in terms of adherence behavior.

The findings should be taken with care due to the sample being representative of three countries; thus, future studies could expand to more countries to examine even more cultural differences. Another limitation is the small sample size for South Korea, limiting the generalizability of the results.

### Conclusions

As the COVID-19 global pandemic continues to grow and governmental restrictions are ongoing, it is critical to understand people’s frustration to reduce panic and promote social distancing to control the pandemic. This study points to a threat and coping appraisal mechanism that may clarify the adherence variations. This study also highlights that social media has an impact on the threat appraisal of the COVID-19 pandemic. Furthermore, the intensity of information sources used to attain COVID-19 information impacts the coping appraisal for the pandemic.

## Appendix

Multimedia Appendix 1 Survey questionnaire and detailed analysis.

## Abbreviations

PMT: protection motivation theory
SUR: seemingly unrelated regression
