# Supplementary material for: Threat, Coping, and Social Distance Adherence During COVID-19: Cross-Continental Comparison Using an Online Cross-Sectional Survey
Source: J Med Internet Res. 2020 Nov 18;22(11):e23019. doi: 10.2196/23019 (PMC7677591; doi:10.2196/23019)
Supplement: Multimedia Appendix 1 [file jmir_v22i11e23019_app1.docx]

**Multimedia Appendix 1**

**Table A1.** Survey questionnaire and coding scheme

| **Variable** | **Description** | **Questionnaire Items***.* | **Refs.** |
| --- | --- | --- | --- |
| *Dependent Variable* | | | |
| ADHERENCE | Individual's intention to adhere to social distancing/sheltering recommendations | 1. I will comply with the sheltering or social distancing measures.  2. Sheltering or social distancing measures are effective at slowing the spread of Covid-19.  3. The government has the right to enforce sheltering (i.e., people must stay at home).  *Scale: 1 - Strongly Disagree to 5-Strongly Agree.*  *Cronbach Alpha: 0.81.* | [23-29] |
| *Independent Variables* | | | |
| X1: THREAT APPRAISAL | Threat appraisal results from and interrelates with the perceived SEVERITY and VULNERABILITY of the COVID-19 pandemic. | | [30] |
| SEVERITY | Perceived severity or degree of harm from engaging in unhealthy behavior; the extent to which one will experience suffer or die from contracting Covid-19 upon ignoring social distancing/sheltering recommendations | 1. My whole family will suffer if I do not follow social distancing or sheltering practices during COVID-19. 2. I will become hopeless if I get infected with COVID-19 because I ignored social distancing or sheltering recommendations. 3. People who go outside during COVID-19 pandemic harms others.  *Scale: 1 - Strongly Disagree to 5-Strongly Agree.*  *Cronbach Alpha: 0.63.* | [23-29] |
| VULNERABILITY | Perceived probability of threat occurrence; the extent to which one will contract Covid-19 upon ignoring social distancing/sheltering recommendations | 1. I will get infected if I go to grocery stores. 2. I will get infected if I go outside without wearing masks. 3. If I use public transportation (i.e., bus, train, airplane), I will get infected.  *Scale: 1 - Strongly Disagree to 5-Strongly Agree.*  *Cronbach Alpha: 0.76.* | [23-29] |
| X2: COPING APPRAISAL | Coping appraisal results from and interrelates with the RESPONSE EFFICACY and SELF EFFICACY of an individual being able to change their health behavior to cope with the COVID-19 pandemic. | | [30] |
| RESPONSE EFFICACY | Perceived efficacy of recommended behavior; beliefs that adopting social distancing/sheltering will be effective in reducing the threat of Covid-19 | 1. People will be less likely to get infected if they stay home. 2. People who wear masks are less likely to spread COVID-19. 3. Washing hands is good for reducing infection.  *Scale: 1 - Strongly Disagree to 5-Strongly Agree.*  *Cronbach Alpha: 0.80.* | [23-29] |
| SELF EFFICACY | Perceived self-efficacy; belief that one can successfully maintain a safe distance from others when in contact or stay home to avoid all contacts | 1. I can keep the masks on even if people around me do not wear one. 2. Even if all who around me stay inside, that does not mean I should stop going outside. 3. I can refuse even if a relative or friend asks me for a hug.  *Scale: 1 - Strongly Disagree to 5-Strongly Agree.*  *Cronbach Alpha: 0.60.* | [23-29] |
| X4: COVID-19  INFORMATION SOURCES | The extent to which an individual uses health information sources to attain COVID-19 information. | Which of the following do you use for Covid-19 related information? Check all that apply (a) Social Media, (b) TV, (c) Newspaper (online and print), (d) Friends or Family, (e) Doctors or Medical Professionals.  *Count of total number of information sources used was used to code the COVID-19 INFORMATION SOURCE variable.* |  |
| X5: COVID-19 SOCIAL MEDIA | The extent to which an individual uses social media to attain COVID-19 information. | Which of the following social media do you use for Covid-19 news? Check all that apply (a) Facebook, (b) Twitter, (c) WhatsApp, (d) Instagram, (e) LinkedIn, (f) Snapchat, (g) TikTok, (h) YouTube, (i) Other.  *Count of total number of social media platforms used to attain COVID-19 information was used to code the COVID-19 SOCIAL MEDIA variable.* |  |
| X6:  KNOWLEDGE | The extent to which one is aware or knowledgeable about COVID-19 and relevant situations. | \| 1- How long does it take for Covid-19  symptoms to appear after exposure?  *(a) 1-2 days, (b) 3-5 days,*  *(c) about a week, (d) about two weeks,*  *(e) more than two weeks* \| \| --- \| \| 2- What are the symptoms of Covid-19?  *Check all that apply (a) Fever, (b) Cough,*  *(c) Difficulty breathing, (d) Nausea,*  *(e) Diarrhea, (f) Skin Rash,*  *(g) Loss of smell and taste,*  *(h) I don't know* \| \| 3- Which of the following characteristics  are associated with high-risk groups?  *Check all that apply (a) Elderly,*  *(b) Diabetes, (c) Smokers, (d) Female,*  *(e) Other* \| \| 4- Can you catch Covid-19 from being  exposed to someone who died of Covid-19?  *(a) Yes, (b) No, (c) I don't know* \| \| 4- Can you catch Covid-19 from being  exposed to pets?  *(a) Yes, (b) No, (c) I don't know* \| \| 5- What is the treatment of Covid-19?  *(a) Drink fluid and getting rest,*  *(b) Hydroxychloroquine,*  *(c) Herbal medicine, (d) Vaccines,*  *(e) None* \| \| 6- If you recover from Covid-19, how  likely will you get sick from future  exposure?  *(a) Never, you are immune for life,*  *(b) Not likely, you are immune for*  *a year or so,*  *(c) Somewhat likely, may get sick but*  *quickly recover,*  *(d) Likely, will get sick again,*  *(e) I don't know* \| \| 7- What do you think is the best method  of decreasing the spread of Covid-19?  *(a) wearing masks, (b) quarantining people,*  *(c) shutting down borders, highways,*  *to restrict travel (d) buying more*  *ventilators, (e) let high-risk people die*  *to achieve herd community* \| \| 8- Do you believe there is adequate testing  to detect the spread of Covid-19?  *(a) Yes, (b) No, (c) I don't know* \| \| 9- How many lives do you believe will be  lost due to Covid-19?  *(a) 10,000's, (b) 100,000's,*  *(c) About 1 Million, (d) 1-10 Million,*  *(e) More than 10 Million* \|   *The aggregated and standardized items* *were taken to code the* KNOWLEDGE *variable.* | [31] |
| X7:  COUNTRY | Country of residence | I reside in: (a) the United States, (b) South Korea, (c) Kuwait.  *Dummy variables were coded for countries based on responses.* |  |
| *Control Variables* | | | |
| C1:  AGE | Age of respondent | My age group is: (a) 18-27 years old, (b) 28-37 years old, (c) 38-47 years old, (d) 48-57 years old, (e) greater than 58 years old  *The variable was coded by taking the responses to the question, where the checked responses to the ordered category choice sets (i.e., five) were transformed into corresponding numerical values.* | |
| C2:  GENDER | Gender of respondent | I am: (a) Male, (b) Female  *The variable was coded by Female = 1 and Male = 0.* | |
| C3:  INCOME | Household income of the respondent | My household income is: (a) Less than $30,000, (b) $30,000 - $50,000, (c) $50,000 - $80,000, (d) $80,000 - $100,000, (e) $100,000 - $150,000, (f) Higher than $150,000  *The variable was coded by taking the responses to the question, where the checked responses to the ordered category choice sets (i.e. six) were transformed into corresponding numerical values.* | |

**Table A2.** Summary statistics of key variables (N=418).

| **Variable** | **Frequency** | **Percentage** |
| --- | --- | --- |
| AGE GROUP - 18-27 | 192 | 37.28 |
| AGE GROUP - 28-37 | 150 | 29.13 |
| AGE GROUP - 38-47 | 70 | 13.59 |
| AGE GROUP - 48-57 | 43 | 8.35 |
| AGE GROUP - 58+ | 60 | 11.65 |
| FEMALE | 299 | 58.74 |
| HOUSEHOLD INCOME - Less than $30K | 102 | 20.52 |
| HOUSEHOLD INCOME - $30K-$50K | 92 | 18.51 |
| HOUSEHOLD INCOME - $50K - $80K | 102 | 20.52 |
| HOUSEHOLD INCOME - $80K-$100K | 65 | 13.08 |
| HOUSEHOLD INCOME - $100K-$150K | 57 | 11.47 |
| HOUSEHOLD INCOME - $150K or more | 79 | 15.90 |
| COUNTRY – KOREA | 71 | 16.99 |
| COUNTRY – US | 162 | 38.76 |
| COUNTRY – KUWAIT | 185 | 44.26 |


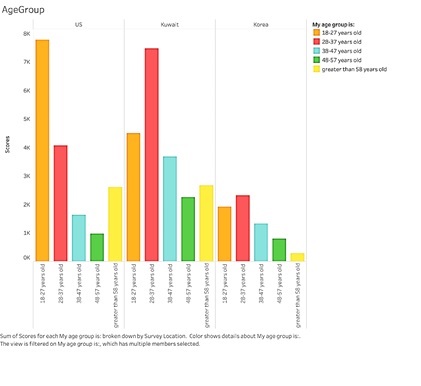


Figure A1. Age groups distribution by country.

**
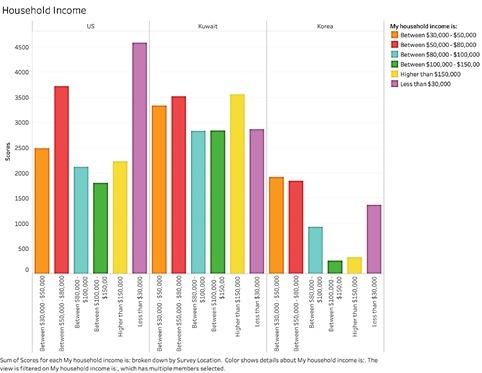
**

Figure A2. Household income distribution by country.

Figure A3. Mean adherence by country.

Figure A4. Mean COVID-19 knowledge by country.

**Table A3.** Ordered Probit regression on detailed PMT variables.

|  | **DV: ADHERENCE** | | | |
| --- | --- | --- | --- | --- |
| **VARIABLES** | (1) | (2) | (3) | (4) |
|  | ALL | US | SOUTH KOREA | KUWAIT |
| SEVERITY | 0.358***  (0.07) | 0.259*  (0.12) | 0.771***  (0.22) | 0.323**  (0.11) |
| VULNERABILITY | -0.066  (0.07) | -0.052  (0.10) | 0.216  (0.19) | -0.122  (0.11) |
| SELF EFFICACY | 0.131*  (0.06) | -0.097  (0.09) | -0.214  (0.25) | 0.390**  (0.12) |
| RESPONSE EFFICACY | 0.627***  (0.08) | 0.701***  (0.13) | 0.692***  (0.18) | 0.454**  (0.17) |
| KNOWLEDGE | 0.047**  (0.02) | 0.016  (0.03) | 0.027  (0.04) | 0.063*  (0.03) |
| AGE | -0.019  (0.04) | -0.035  (0.06) | -0.188  (0.14) | -0.007  (0.07) |
| GENDER(FEMALE) | 0.117  (0.11) | 0.181  (0.18) | 0.329  (0.28) | 0.103  (0.18) |
| HOUSEHOLD_INCOME | 0.010  (0.03) | 0.066  (0.06) | -0.115  (0.11) | -0.071  (0.05) |
| Observations | 418 | 162 | 71 | 185 |
| Pseudo R-sq. | 0.1338 | 0.0937 | 0.2677 | 0.0826 |
| Chi. sq. | 225.92 | 63.01 | 89.80 | 46.47 |

^Standard errors in parentheses.*^ *^P^* ^< 0.05, **^ *^P^* ^< 0.01, ***^ *^P^* ^< 0.001.^

**Table A4.** Comparison of coefficients across countries on detailed PMT variables.

|  | **DV: ADHERENCE** | | |
| --- | --- | --- | --- |
| **Variables** | US vs. Kuwait | US vs.  South Korea | South Korea vs. Kuwait |
| SEVERITY | 0.14 | 5.57** | 3.98** |
| VULNERABILITY | 0.24 | 2.24 | 3.35* |
| SELF EFFICACY | 8.76*** | 0.18 | 4.32** |
| RESPONSE EFFICACY | 1.15 | 0.00 | 1.14 |
| KNOWLEDGE | 1.27 | 0.05 | 0.68 |

^(Chi. sq. values reported with Bonferroni-adjustment, *^ *^P^* ^< 0.05, **^ *^P^* ^< 0.01, ***^ *^P^* ^< 0.001)^

**Table A5.** SUR on social media categories (social network, media sharing, and text/microblogging)

|  | **DV: COPING APPRAISAL** | | | | | **DV: THREAT APPRAISAL** | | | | | **DV: ADHERENCE** | | | | |
| --- | --- | --- | --- | --- | --- | --- | --- | --- | --- | --- | --- | --- | --- | --- | --- |
| **VARIABLES** | (1) | (2) | (3) | (4) | (5) | | (6) | (7) | (8) | (9) | | (10) | (11) | (12) |  |
|  | ALL | US | SOUTH KOREA | KUWAIT | ALL | | US | SOUTH KOREA | KUWAIT | ALL | | US | SOUTH KOREA | KUWAIT |  |
| COVID-19 INFO. SORC. | 0.132***  (0.03) | 0.0918*  (0.04) | 0.211**  (0.08) | 0.057  (0.04) | 0.047  (0.04) | | 0.026  (0.05) | 0.205  (0.12) | 0.081  (0.05) |  | |  |  |  |  |
| COVID-19 SOC. NET. | -0.228**  (0.07) | 0.160  (0.09) | 0.071  (0.17) | -0.072  (0.11) | -0.063  (0.08) | | 0.254*  (0.12) | -0.124  (0.25) | -0.066  (0.14) |  | |  |  |  |  |
| COVID-19 MED. SHRNG. | -0.021  (0.04) | -0.071  (0.06) | 0.007  (0.11) | 0.009  (0.05) | 0.084  (0.05) | | 0.070  (0.08) | -0.063  (0.17) | 0.030  (0.06) |  | |  |  |  |  |
| COVID-19 MICROBLG. | 0.249***  (0.05) | -0.026  (0.10) | -0.246  (0.16) | -0.016  (0.06) | 0.240***  (0.06) | | 0.341**  (0.13) | -0.286  (0.23) | 0.006  (0.07) |  | |  |  |  |  |
| THREAT APPRAISAL |  |  |  |  |  | |  |  |  | 0.214***  (0.05) | | 0.212**  (0.08) | 0.585***  (0.12) | 0.048  (0.08) |  |
| COPING APPRAISAL |  |  |  |  |  | |  |  |  | 0.501***  (0.06) | | 0.328**  (0.11) | 0.506**  (0.16) | 0.533***  (0.11) |  |
| KNOWLEDGE |  |  |  |  |  | |  |  |  | 0.061***  (0.01) | | 0.053*  (0.02) | 0.048  (0.03) | 0.040*  (0.02) |  |
| AGE | -0.001  (0.03) | -0.101**  (0.04) | -0.015  (0.08) | 0.047  (0.03) | 0.017  (0.03) | | -0.043  (0.05) | 0.031  (0.11) | 0.053  (0.04) | 0.006  (0.03) | | 0.037  (0.05) | -0.012  (0.08) | -0.038  (0.04) |  |
| FEMALE | 0.232**  (0.07) | 0.007  (0.11) | 0.055  (0.18) | 0.085  (0.08) | 0.185*  (0.08) | | 0.084  (0.14) | -0.132  (0.26) | 0.236*  (0.11) | 0.041  (0.08) | | -0.024  (0.14) | 0.206  (0.17) | 0.044  (0.12) |  |
| HOUSEHOLD  INCOME | 0.0460*  (0.02) | -0.032  (0.03) | 0.014  (0.07) | 0.029  (0.02) | -0.0428  (0.03) | | -0.0889*  (0.04) | 0.0425  (0.10) | -0.0548  (0.03) | -0.001  (0.02) | | 0.012  (0.04) | -0.044  (0.06) | -0.011  (0.03) |  |
| constant | 2.260***  (0.13) | 2.676***  (0.18) | 1.798***  (0.29) | 3.033***  (0.15) | 2.895***  (0.15) | | 2.847***  (0.24) | 2.922***  (0.43) | 3.198***  (0.19) | 1.061***  (0.23) | | 1.700***  (0.45) | -0.113  (0.39) | 1.989***  (0.52) |  |
| Observations | 418 | 162 | 71 | 185 | 418 | | 162 | 71 | 185 | 418 | | 162 | 71 | 185 |  |
| R-sq. | 0.1586 | 0.0979 | 0.1480 | 0.0498 | 0.0771 | | 0.1553 | 0.0598 | 0.0647 | 0.3744 | | 0.1793 | 0.6688 | 0.1584 |  |
| Chi. sq. | 78.84 | 17.59 | 12.34 | 9.75 | 35.04 | | 30.13 | 4.54 | 12.82 | 244.41 | | 33.51 | 144.50 | 33.19 |  |
| P-value | 0.0000 | 0.0139 | 0.0899 | 0.2034 | 0.0000 | | 0.0001 | 0.7159 | 0.0766 | 0.0000 | | 0.0000 | 0.0000 | 0.0000 |  |

^Standard errors in parentheses.*^ *^P^* ^< 0.05, **^ *^P^* ^< 0.01, ***^ *^P^* ^< 0.001.^

**Table A6.** Summary of findings on detailed PMT and social media platform constructs

| **Variables** | **All** | **US** | **South Korea** | **Kuwait** | |  |
| --- | --- | --- | --- | --- | --- | --- |
|  |  | | | | **Findings** | |
|  | **Adherence** | | | |  | |
| SEVERITY | + | + | + | + | Severity positively influences social distancing adherence. Severity is more influential in the US than in South Korea; and more influential in South Korea than in Kuwait. | |
| VULNERABILITY | NS | NS | NS | NS | Vulnerability does not significantly influence adherence. Vulnerability is more influential in South Korea than in Kuwait. | |
| SELF EFFICACY | + | NS | NS | + | Self Efficacy positively influences social distancing adherence in the whole sample and in Kuwait. Self Efficacy is more influential in the US and South Korea than in Kuwait. | |
| RESPONSE EFFICACY | + | + | + | + | Response efficacy positively influences social distancing adherence. No comparative difference in results across countries. | |
|  | **Threat Appraisal** | | | |  | |
| COVID-19 SOCIAL NETWORK | NS | + | NS | NS | Using social network platforms for COVID-19 information positively influences threat appraisal in the US. | |
| COVID-19 MEDIA SHARING | NS | NS | NS | NS | No significance of Media Sharing platforms. | |
| COVID-19 TEXT/  MICROBLOGGING | + | + | NS | NS | Using text/microblogging platforms for COVID-19 information positively influences threat appraisal in the whole sample and the US. | |
|  | **Coping Appraisal** | | | |  | |
| COVID-19 SOCIAL NETWORK | - | NS | NS | NS | Using social network platforms for COVID-19 information negatively influences coping appraisal in the US. | |
| COVID-19 MEDIA SHARING | NS | NS | NS | NS | No significance of Media Sharing platforms. | |
| COVID-19 TEXT/BLOGGING | + | NS | NS | NS | Using text/microblogging platforms for COVID-19 information positively influences coping appraisal in the whole sample. | |

^NS= Not Significant.^
